# GIS-based precise predictive model of mountain beacon sites in Wenzhou, China

**DOI:** 10.1038/s41598-022-15067-z

**Published:** 2022-06-24

**Authors:** Lifeng Tan, Bei Wu, Yukun Zhang, Shuaishuai Zhao

**Affiliations:** 1 School of Architecture, Tianjin Chengjian University, Tianjin, China; 2 Key Laboratory of Information Technology for Architectural Heritage Inheritance of the Ministry of Culture and Tourism of China, School of Architecture, Tianjin University, Tianjin, China

**Keywords:** Computer science, Information technology

## Abstract

In ancient China, where was frequently troubled by invaders, the government set up many beacon towers for alerting and transmitting military information along the border and the coast. Many beacon sites still exist in some areas, which are generally located in dangerous places with high mountains and rough terrain, bringing great difficulties to archaeological discovery. Therefore, it is particularly important to develop a predictive model applicable to the distribution of mountain beacon sites. Taking 68 beacon sites found in Wenzhou as research samples, this study used the superimposed method of logistic regression and viewshed analysis, forming a high-precision, scientific and operational predictive model for the distribution of beacon sites, which was verified by the cross-validation method. The results showed that the beacon site predictive model simulated in this study could reduce the probability scope of site location by 90% compared with the common logistic regression predictive model, which greatly improved the accuracy and ability of site prediction. At the same time, it could also be used to understand the relationship between the known sites and their surroundings to assist in decision-making about conservation and management.

## Introduction

Cultural heritage, as a witness of history, is a precious treasure left to mankind by history. In the coastal areas of China, there are many beacon tower remains, which were high platforms used to light fires to convey messages in ancient times, built to prevent enemy invasion. Beacon tower (烽燧, Feng Sui), the fastest and most effective way to transmit military information in the cold weapon era, has a long history in China^1^. According to the Records of the Grand Historian, there were beacon towers as early as the Western Zhou Dynasty, and the beacon system was quite mature during the Han Dynasty and reached its peak during the Tang Dynasty, following which it was used throughout the Song to Qing Dynasties^2^. Before the emergence of modern communication technology, beacon towers had been valued by successive Chinese dynasties as ancient but effective military communication tools and were commonly placed in border areas. In the Ming Dynasty, the coastal areas were constantly invaded by remnant rebel forces and Japanese pirates, and the maritime situation was extremely severe. To anticipate the invasion of enemies and take timely precautions, the Ming government constructed beacon towers along the coast as indispensable coastal defense facilities to resist enemies. When enemies occurred, smoke was burnt during the day, called "Feng", and fire was lit at night, called "Sui", so that the beacon towers were connected to convey information^3^. Once the enemy appeared, the garrisons of the beacon towers would quickly light the fire to report important military information so that the message of invasion could be spread one by one at the fastest speed until the soldiers in the nearby forts found it. Beacon towers, together with forts, played a vital role in the struggle against Japanese pirates on the coastline. Therefore, a systematic study on the distribution of beacon towers is a prerequisite and necessary process for the exploration of coastal defense systems. To obtain a good view and expand its coverage as much as possible, beacon towers were usually built-in places with high altitudes and wide vision. Therefore, there are many beacon sites on the mountaintops and high hills in coastal areas. However, over the course of history for centuries, beacon sites have gradually faced extinction due to natural erosion and man-made vandalism. Thus, it is exceedingly significant to discover these beacon sites as soon as possible and implement scientific and effective conservation measures.

However, the discovery of mountain beacon sites is time consuming and labor consuming. Owing to their long history, remote location, vegetation coverage, dilapidated condition and small dimension, beacon sites are difficult to investigate so that archaeologists usually acquire site information by asking local residents, consulting materials and combining field investigations. However, in fact, few local residents truly know about the beacon sites, so the useful information that can be obtained through inquiry is very limited, which poses great difficulties to archaeologists. According to the local archaeologists, the mountainous terrain in Wenzhou was complex and rugged, with tight vegetation coverage. In most cases, they could not rely on transportation tools, and the only way to reach the mountaintops was by walking. In extreme weather conditions, there are also many potential dangers. Even after overcoming those physical difficulties, there was no guarantee that new beacon sites would be found in each survey, which greatly reduced the efficiency of archaeological work and was very detrimental to conservation work. Therefore, we intend to simulate a predictive method for the distribution probability of mountain beacon sites to solve these difficulties, reduce the research workload, and improve the efficiency of the site survey.

Site probability prediction based on the assumption that the distribution of sites was not random but was related to specific characteristics of the natural environment and factors associated with human activity and norms of human behavior in the past^4^. Over the years, the most commonly used method of site probability prediction has been to build a mathematical model in the research area and calculate the probability values that the site may exist in an unknown area by analyzing the data of known samples^5^. This research originated in the 1950s, when Willey (1953) first applied the predictive model to the regional settlements of Weilu Valley, providing insight into the relationship between site and environment through qualitative analysis^6^. In the 1970s, quantitative analysis emerged, and Green (1973) used multiple linear regression to predict the probability of the existence of prehistoric Mayan sites in Northern British Honduras, laying the groundwork for the subsequent application of site predictive models to cultural resource management^7^. Thanks to the development of computer science, the two disciplines of statistics and spatial analysis skillfully combined in the 1980s led to great advances in site predictive research. Kvamme (1983) was the main advocate of this technique, and he proposed an approach for regional modeling of archaeological site locations based on computer processing techniques applied to digital elevation data^8^. Subsequently, books written by Kohler (1988), Judge & Sebastian (1988) and Kvamme (1988) were published, which were highly influential at that time^9–11^. Entering the 1990s, with the emergence of relevant computer software and the continuous promotion of Geographic Information Systems (GIS) in the field of archaeological research, the research methodologies gradually diversified. Märker & Heydari-Guran (2009) used data-mining techniques to predict Paleolithic site locations in the Zagros Mountains of Iran^12^. Casarotto, et al. (2011) proposed a predictive model to simulate an ancient landscape in the Eastern Lessinia by exploiting GIS suitability maps and multi-criteria target analysis^13^. Dorothy Graves (2011) developed a site predictive modelling using Logistic Regression method to target Neolithic settlement and occupation activity in mainland Scotland^14^. Scientific and sophisticated predictive means had vigorously developed site predictive research in terms of both depth and breadth^15–18^.

Constructing a site predictive model is a complex task that requires effective methodology, appropriate parameters, and a scientific approach of validation^19^. Statistical regularities and distribution characteristics of known sites are identified first based on the analysis of environmental factors in the given area, such as altitude, slope, distance from transport system or hydrographic net; then, a multivariate discriminant function is adopted to assess the existence probability of other sites in this area, giving a potential site distribution map^20^. The application of quantitative analysis makes the study of human-land relationships more accurate and scientific, among which the most widely used is the logistic regression analysis method^21^. This approach is frequently conducted as a useful tool for archaeological investigations, not only providing an important decision support system to obtain usable information but also saving time and money, especially in large areas^21^. Based on the previous research, this study will construct a precise predictive model applicable to the mountain beacon sites by identifying the causal relationship between certain environmental characteristics, human activities and known sites.

## Materials and methods

### Study area

**Figure 1:**
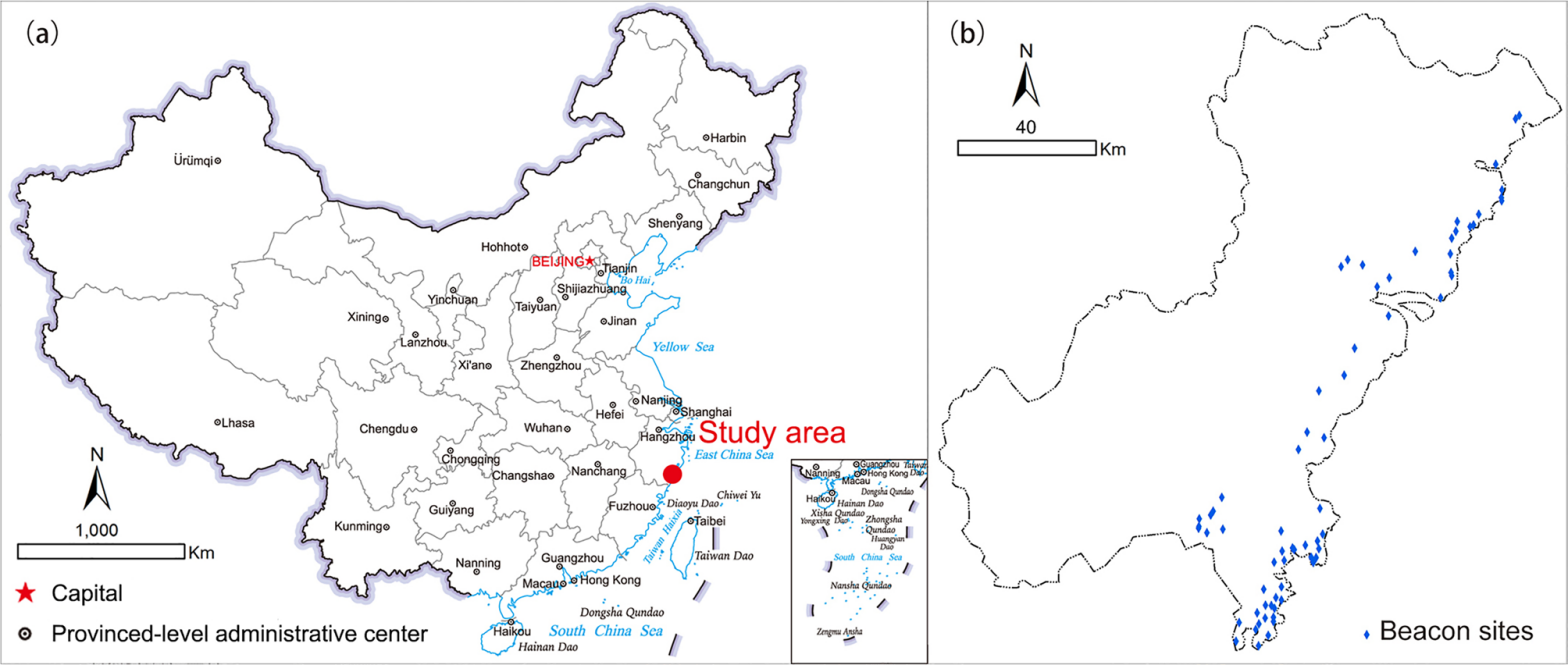
Study area: (**a**) location of Wenzhou (provided by Ministry of Natural Resources of China, Approval number: GS(2019)1673); (**b**) distribution of beacon sites (generated by the authors using ArcGIS 10.3.1).

The Wenzhou (温州) area was chosen as the study area, including the inner city of Wenzhou, as well as the counties and villages under its jurisdiction, with a total area of approximately 12,110 km^2^^22^. Wenzhou is located in East China, southeast of Zhejiang Province, lower reaches of the Ou River, bordering the East China Sea to the east and Fujian Province to the south, which is one of the nationally famous historical and cultural cities (Fig. 1a). Being a coastal city, Wenzhou had been a key place for military defense throughout its history. Especially in the Ming Dynasty, Wenzhou was heavily infested by Japanese pirates with an extremely serious situation, so 58 beacons were built in Yueqing County, Rui'an County and Pingyang County along the coast of Wenzhou to prevent and resist enemies and were increased in the late Ming and Qing Dynasties^23^. By far, archaeologists have discovered 68 beacon sites in the Wenzhou area (Fig. 1b), most of which have fallen into disrepair. The materials used to build the beacon towers were all taken from nature, mostly made of clay and gravel, and rammed layer by layer; some were piled up with bricks and stones, with square, rectangular and round shapes. These beacon sites are historical evidence of Zhejiang coastal people's bravery in fighting against Japanese pirates. Based on the known data, this study will simulate a predictive method applicable to Wenzhou’s mountain beacon sites.

### Methodology

**Figure 2:**
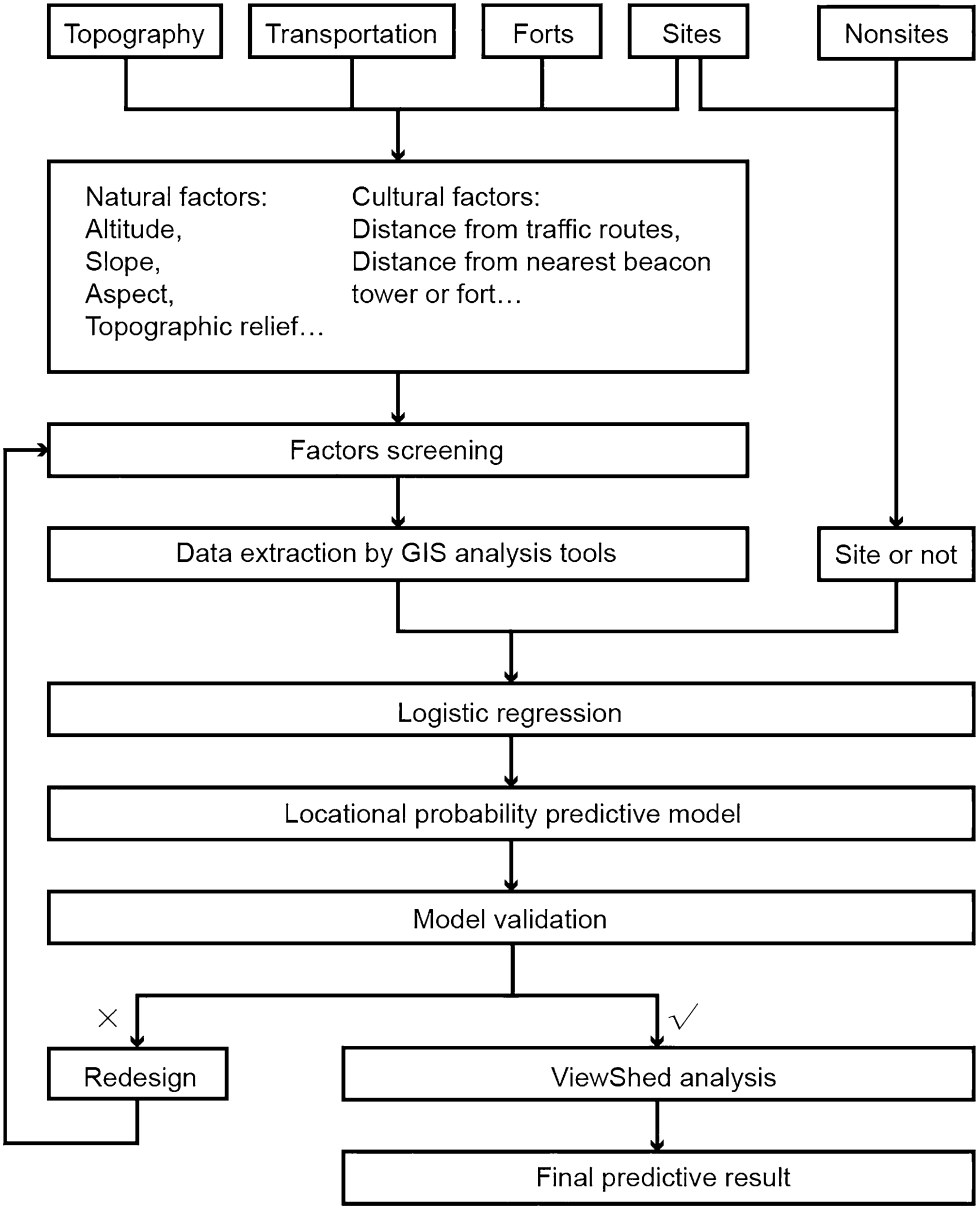
Working flow chart for the prediction of beacon sites in Wenzhou.

The site selection of mountain beacon towers was not random, but was a result of logical decisions based on geographical considerations and factors related to human activity. Most of them were found on the mountaintops along the traffic arteries between the forts or on the high hills of the coastal peninsula, where were easy to look out. Moreover, due to the requirement of visual factors, beacon towers were placed to ensure the accessibility of sightlines. Considering the two factors of geography and vision, we proposed to adopt the superimposed method of logistic regression and GIS viewshed analysis to simulate beacon sites’ location preferences. Theoretically, this method can effectively improve the prediction accuracy and further narrow the possible scope of unknown beacon sites compared to the single logistic regression predictive method commonly used in the past. The working flow pursued the following steps (Fig. 2): (1) The historical construction characteristics and distribution features of known beacon sites were analyzed to screen out the natural and cultural factors affecting their positions in space; (2) logistic regression analysis was integrally conducted on the beacon sites, *nonsites* (a concept introduced to represent locations where sites were not found) and multiple independent variables, establishing a GIS calculation model for predicting the discovery probability of unknown sites in the given area; (3) the accuracy of the result was verified by the cross-validation method; and (4) GIS viewshed analysis was superimposed on the previous result to obtain the final prediction result of unknown beacon sites.

#### Data sources and extraction

The data used to build the predictive model of mountain beacon sites consisted of two groups: the experimental samples (dependent variables) and the environmental factors (independent variables). First, 168 experimental samples in the survey area were required to study the probability of the dependent variables, including 68 sites and 100 *nonsites*. The number of experimental samples depends on the size of the study area, usually making the spatial distribution density of sites and *nonsites* equal. The data of beacon sites were collected from the survey data of the Wenzhou Institute of Cultural Relics and Archaeology (Fig. 1b), and the *nonsites* were randomly introduced specifically for the predictive model. Conceptually, all the locations where sites were not found can be used as *nonsites*, so the "Create Random Points" tool of ArcGIS software was used to generate 100 random points as *nonsites* of the experimental samples. In this study, the experimental samples consisted of 68 discovered beacon sites, and 100 randomly acquired *nonsites*.

The independent variables related to the construction of the predictive model required to be screened out. For the independent variables, natural and cultural factors such as altitude, slope, distance, etc., that potentially influenced the spatial distribution of the sites, were integrated into the predictive model. The selection of independent variables was mainly determined by the mode and features of beacon transmission during the Ming and Qing dynasties, combined with the distribution characteristics of existing sites.

Wenzhou is mostly mountainous, and the terrain generally shows the features of high inland and low coastal areas; hence, the topographical parameters were mainly considered for natural variables. The survey data showed that the discovered beacon sites were mainly located in the coastal area and on the top of the hill with a good view to facilitate the information transmission among beacon towers. In addition, considering the delivery of living supplies and smoke-burning materials, the locational selection of the beacon towers must also consider the easy access. Therefore, the altitude, slope, aspect and topographic relief, that may be linked to their locations, were taken as alternative independent variables for further screening.

Except for topographical factors, the distribution of beacon sites was also probably influenced by cultural factors related to human activity. For example, because the beacon tower relied on smoke to transmit, only the neighboring beacon tower or fort was set within its viewing range so that the information could be transmitted one by one until the soldiers of forts received it, so the distance from nearest beacon tower or fort was inevitably under consideration when constructing the beacon system. Similarly, due to the need for convenient access to materials and resources, the distance between beacon towers and main traffic routes might also be one of the factors to be measured. Therefore, the cultural variables considered in this study mainly included two variables, namely, the distance from traffic routes and the distance from the nearest beacon tower or fort.

**Figure 3:**
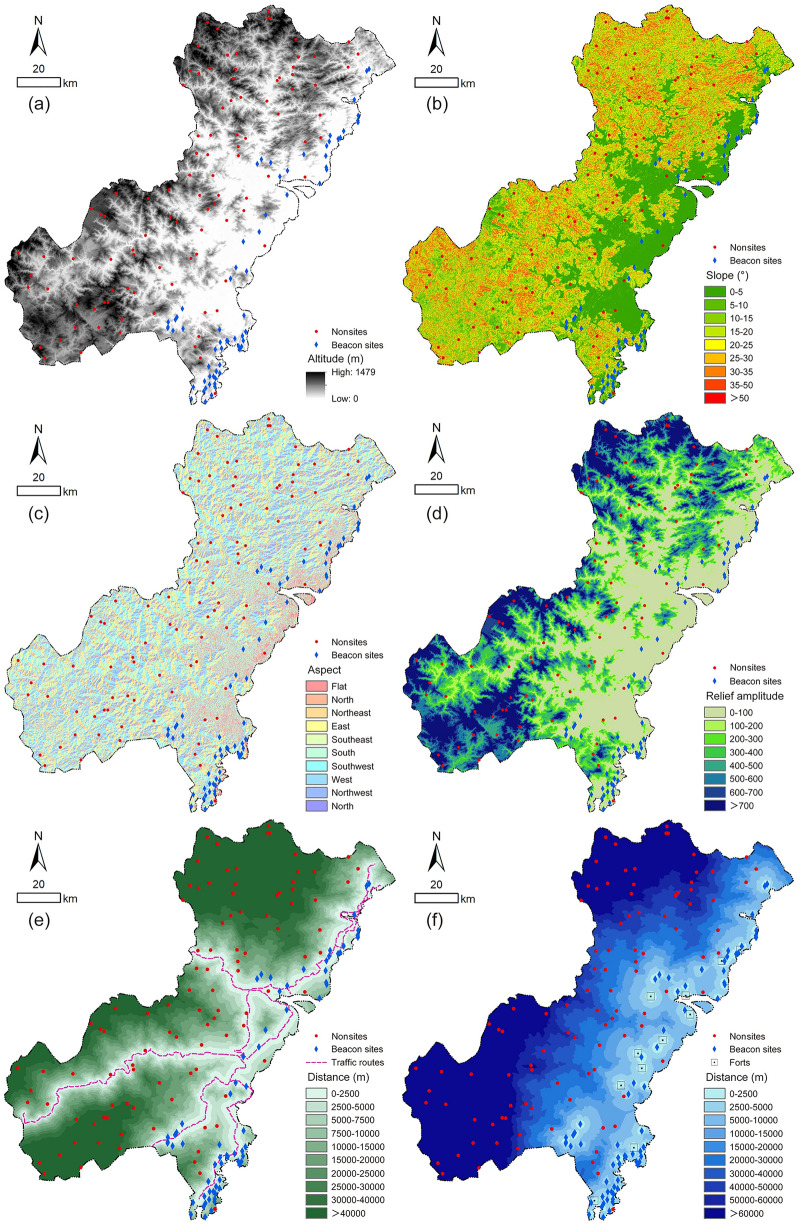
Raster data of alternative independent variables: (**a**) altitude; (**b**) slope; (**c**) aspect; (**d**) topographic relief; (**e**) distance from traffic routes; (**f**) distance from nearest beacon tower or fort (generated by the authors using ArcGIS 10.3.1).

In general, six alternative independent variables were selected in this study for further screening, and the relevant data could be extracted using GIS spatial analysis tools (Table 1). Variables related to natural factors, including altitude, slope, aspect and topographic relief, were extracted from the Digital Elevation Model (DEM) data of the Wenzhou area, provided by the Geospatial Data Cloud site, Computer Network Information Center, Chinese Academy of Sciences (http://www.gscloud.cn); variables linked to cultural factors, including the distance from traffic routes and nearest beacon tower or fort, were derived from the historical literature^23^. By superimposing the vector data of traffic routes, beacon towers and forts data with DEM data, extracting through GIS analysis tools, the raster data of each alternative independent variable required in this study were obtained (Fig. 3).

**Table 1:** Alternative independent variables.

Type	Independent variables	Data sources	Spatial resolution (m)	Coordinate system	Calculation method
Natural factors	Altitude	DEM	28.07, 28.07	Geographic coordinate system: GCS_WGS_1984 Projected coordinate system: WGS_1984_UTM_Zone_50N	–
	Slope				ArcGIS → slope
	Aspect				ArcGIS → aspect
	Topographic relief				ArcGIS → block statistics
Cultural factors	Distance from traffic routes	Routes of urgent delivery stations in the Ming Dynasty^23^			ArcGIS → cost distance
	Distance from nearest beacon tower or fort	Beacon sites data, military forts data in the Ming Dynasty^23^			ArcGIS → near analysis

Topographic relief and slope were the main two factors considered in the calculation of cost distance, and the equation used to calculate the cost distance was “cost distance = topographic relief × 0.4 + slope × 0.6”^24^.

#### Independent variables screening

In the second stage, the alternative independent variables were screened by statistically analyzing the degree of correlation between beacon sites, *nonsites* and each alternative environmental variable, and those that are significantly correlated with the locational choice of beacon sites, were considered as the independent variables. For the five factors of altitude, slope, aspect, topographic relief and distance from traffic routes, superimposing the raster data with the distribution map, the attribute values of all the 68 beacon sites and 100 *nonsites* were extracted using the "Extract Multi Values to Points" tool of ArcGIS software. The distance from the nearest beacon tower or fort was calculated by the "Near Analysis" tool of ArcGIS software (Supplementary Table S1 and S2). The results obtained were analyzed by Bulked Segregant Analysis method^25^. If the data of an alternative independent variable showed obvious aggregation at the sites and random or opposite distribution at the *nonsites*, the factor was significantly associated with the locational choice of beacon sites and was screened as the independent variable of the study.

#### Logistic regression and modeling

After identifying the dependent and independent variables, logistic regression analysis was carried out with the Statistical Product and Service Solutions (SPSS) software. Logistic regression is a nonlinear categorical statistical method used in regression analysis of qualitative variables, and later popularized and widely used in population statistics and prediction^26^. According to the differences in dependent variables, logistic regression can be divided into binary logistic regression and multinomial logistic regression. Binary logistic regression, in which the dependent variable can only take values 0 and 1, has the advantage of dealing with multiple types of independent variables such as classification, sequencing and distance fixing at the same time, which is suitable for analyzing complex variables and the interactions among them^27^. Multinomial logistic regression has three or more dependent variables, which can be regarded as several binary logistic regressions and is suitable for complex cases with multiple dependent variables^28^. In this study, the dependent variables were only *nonsites* and sites, i.e. 0 and 1, so the binary logistic regression method was adopted to quantitatively analyze the locational preferences of beacon sites in the Wenzhou area.

According to the rule of binary logistic regression modeling, the existing probabilities of beacon sites were translated using a sigmoid cost function to values in the range of [0, 1], as presented in Eq. (1)^29^.

1 $$\mathrm{P}=\frac{{\mathrm{e}}^{\mathrm{z}}}{1+{\mathrm{e}}^{\mathrm{z}}}$$

where P is the probabilities of the existence of beacon sites; z is the linear combination, obtained from Eq. (2).

2 $$\mathrm{z}=\mathrm{\alpha }+{\upbeta }_{1}{\mathrm{x}}_{1}+{\upbeta }_{2}{\mathrm{x}}_{2}+\cdots .+{\upbeta }_{\mathrm{n}}{\mathrm{x}}_{\mathrm{n}}$$

where α is a constant term, indicating the natural logarithm of the ratio when the independent variable takes the value of 0; ${x}_{n}$ refers to the independent variables affecting the distribution of sites; and ${\beta }_{n}$ is the regression coefficient of the logistic regression.

However, when the number of alternative independent variables is large, the variables may have a high correlativity, and some of them may not have a significant effect on the dependent variable. Hence, the target variables need to be screened for significance in the logistic regression analysis. The "Backward: LR" algorithm of SPSS software can automatically screen and eliminate variables with low significance relationships with the model and carry out iterative calculations to arrive at the ideal model parameters.

With that, a predictive model of the beacon sites was generated using the Map Algebra operation. Map algebra is a high level spatial modeling language that conducts mathematical operations on multiple elements of spatial scope^30^, and can be conducted by the “Map Algebra” tool in the ArcGIS software. Through Eq. (1), a locational probability map of the beacon sites in the Wenzhou area was obtained.

#### Model validation

After establishing the predictive model, the validity and accuracy of the predictive model was evaluated. To meet this requirement, this study used the cross-validation method to evaluate the accuracy of the predictive model. Cross-validation is a statistical method based on the idea of data slicing, in which the whole dataset is split into several datasets according to the actual needs^31^. In this study, we divided the beacon site dataset into two groups, one of which was used as the training sample and the remaining as the validation sample. To ensure the spatial distribution density of training samples and *nonsites* equal, we selected 85% of sites as training samples in this study, that is, 58 sites were taken as training samples, and the remaining 10 sites were used as validation samples. Additionally, considering the homogeneity of the samples and further calculations, the sites in the uniformly distributed areas were selected as validation samples.

#### Viewshed analysis

Having confirmed the validity of the predictive model, a further analysis was then performed on this basis to precisely determine the prediction range. Due to the influence of visual factor, the beacon towers must be set up within the effective visual range of each other, so as to form one or more coherent information flows and serve as a qualified enemy alerting system. Thus, the beacon towers must be in places that not only meet the requirements of terrain and resources but also ensure the safety and no obstruction of the sightline. Based on that, the "Viewshed" tool of ArcGIS software was used for superimposed analysis. The fundamental principle is as follows: assuming there are beacons A and B in a certain area and the sightline is obstructed, it can be concluded that there was a beacon C between A and B that has not yet been discovered. Then, by calculating the viewshed of beacons A and B and superimposing the overlapping area with the high probability area of the sites, we can obtain the possible existence area of beacon C.

Finally, the final prediction result was obtained by superimposing the viewshed analysis result on the distribution probability map of the beacon sites.

## Results and discussion

**Figure 4:**
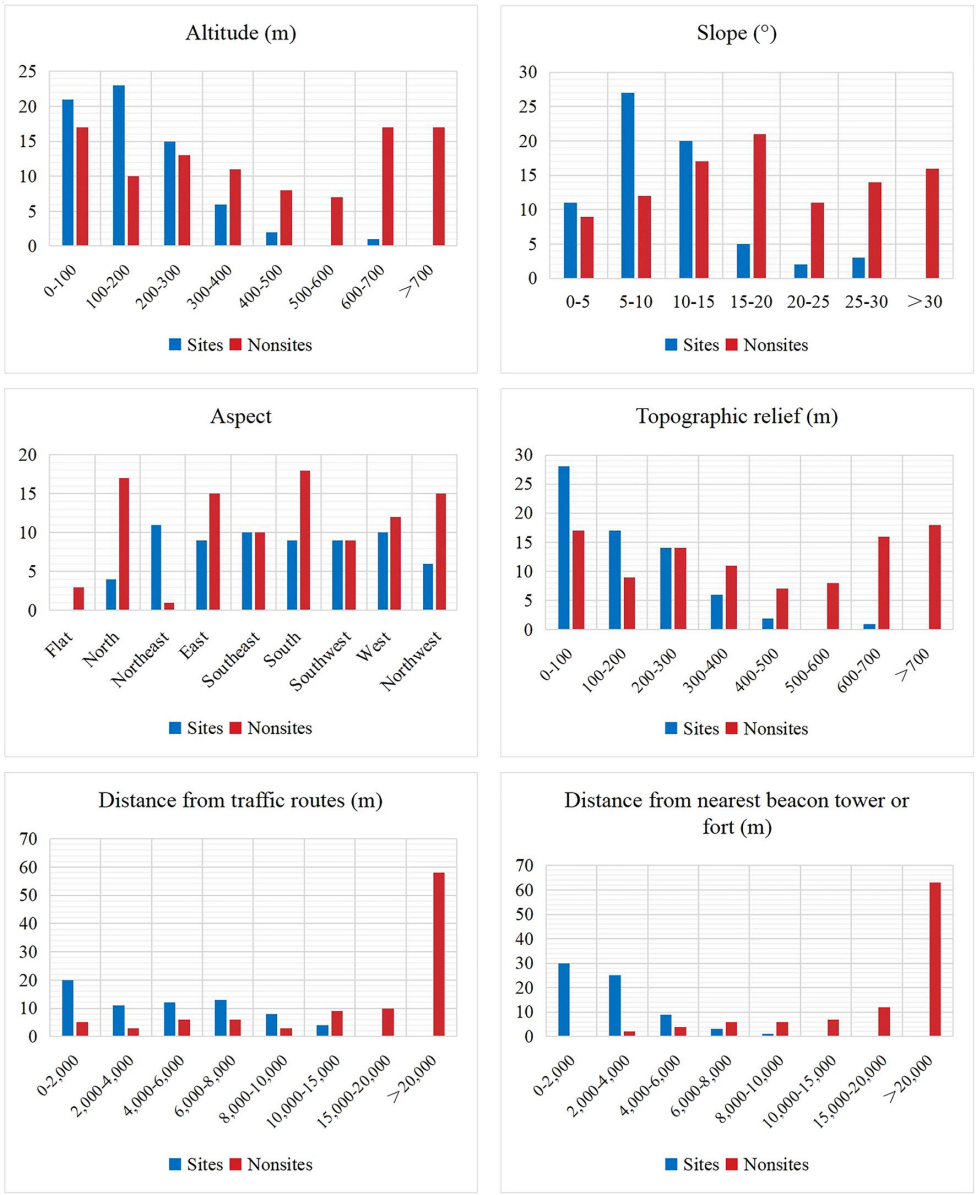
Beacon sites and *nonsites* distribution graphics.

The calculations and analyses were conducted sequentially following the steps in Fig. 2. The statistical results of independent variables screening are shown in Table 2 and Fig. 4. Results showed that the beacon sites locations were preferred in terrain areas with altitude 0–300 m, slope 0–15°, and topographic relief 0–300 m, whereas showing no obvious preference for aspect; the distances from traffic routes are relatively close, mostly reachable within 10,000 m; and the distribution of beacon sites is relatively concentrated, mostly within 6000 m apart. While *nonsites* locations showed a random or opposite distribution pattern compared with sites. This suggested that the locations of beacon sites presented a greater correlation with altitude, slope, topographic relief, distance from traffic routes and nearest beacon tower or fort, while there was no obvious correlation with aspect. Therefore, in this study, altitude, slope, topographic relief, distance from traffic routes and distance from nearest beacon tower or fort were taken as independent variables in the predictive model of beacon sites.

**Table 2:** Beacon sites and *nonsites* distribution statistics.

Factors	Interval partition	Number of sites	Percentage of the total sites (%)	Number of *nonsites*	Percentage of the total *nonsites* (%)
Altitude (m)	0–100	21	30.88	17	17
	100–200	23	33.82	10	10
	200–300	15	22.06	13	13
	300–400	6	8.82	11	11
	400–500	2	2.94	8	8
	500–600	0	0	7	7
	600–700	1	1.47	17	17
	> 700	0	0	17	17
Slope (°)	0–5	11	16.18	9	9
	5–10	27	39.71	12	12
	10–15	20	29.41	17	17
	15–20	5	7.35	21	21
	20–25	2	2.94	11	11
	25–30	3	4.41	14	14
	> 30	0	0	16	16
Aspect	Flat	0	0	3	3
	North	4	5.88	17	17
	Northeast	11	16.18	1	1
	East	9	13.24	15	15
	Southeast	10	14.71	10	10
	South	9	13.24	18	18
	Southwest	9	13.24	9	9
	West	10	14.71	12	12
	Northwest	6	8.82	15	15
Topographic relief (m)	0–100	28	41.18	17	17
	100–200	17	25	9	9
	200–300	14	20.59	14	14
	300–400	6	8.82	11	11
	400–500	2	2.94	7	7
	500–600	0	0	8	8
	600–700	1	1.47	16	16
	> 700	0	0	18	18
Distance from traffic routes (m)	0–2000	20	29.41	5	5
	2000–4000	11	16.18	3	3
	4000–6000	12	17.65	6	6
	6000–8000	13	19.12	6	6
	8000–10,000	8	11.76	3	3
	10,000–15,000	4	5.88	9	9
	15,000–20,000	0	0	10	10
	> 20,000	0	0	58	58
Distance from nearest beacon tower or fort (m)	0–2000	30	44.12	0	0
	2000–4000	25	36.76	2	2
	4000–6000	9	13.24	4	4
	6000–8000	3	4.41	6	6
	8000–10,000	1	1.47	6	6
	10,000–15,000	0	0	7	7
	15,000–20,000	0	0	12	12
	> 20,000	0	0	63	63

With the aim of verifying the accuracy of the predictive model, this study divided beacon sites into two groups, ten sites for validation samples and 58 sites for experimental samples, as detailed in “Model validation”. Extracting data on altitude (X1), slope (X2), topographic relief (X3), distance from traffic routes (X4) and distance from nearest beacon tower or fort (X5) of the 58 beacon sites and 100 *nonsites* in the experimental samples (Supplementary Table S3), a binary logistic regression analysis was conducted (Table 3).

**Table 3:** Predictive model parameters of beacon sites.

	Variables	β	S.E.	Wald	df	Sig	Exp(β)
Step 1	X1	0.258051	0.103580	6.206711	1	0.012727	1.294404
	X2	0.009176	0.043931	0.043627	1	0.834550	1.009218
	X3	−0.252908	0.104060	5.906916	1	0.015082	0.776540
	X4	−0.000343	0.000148	5.342546	1	0.020811	0.999657
	X5	−0.000639	0.000169	14.214303	1	0.000163	0.999362
	Constant	4.768720	1.462610	10.630323	1	0.001112	117.768459
Step 2	X1	0.258674	0.103840	6.205526	1	0.012735	1.295211
	X3	−0.253382	0.104345	5.896675	1	0.015169	0.776171
	X4	−0.000339	0.000147	5.303766	1	0.021279	0.999661
	X5	−0.000640	0.000170	14.209285	1	0.000164	0.999361
	Constant	4.851323	1.424890	11.591977	1	0.000662	127.909457

*β* regression coefficient. *S.E.* standard error, *Wald* value used to test whether the independent variable has an impact on the dependent variable, *df* degree of freedom, *Sig.* significance level, *Exp(β)* odds ratio^32^.

The calculation result indicated that although slope was one of the influencing factors of beacon tower distribution, the significance was not high in the model. Therefore, the final predictive model contained four independent variables, namely, altitude (X1), topographic relief (X3), distance from traffic routes (X4) and distance from nearest beacon tower or fort (X5).

**Figure 5:**
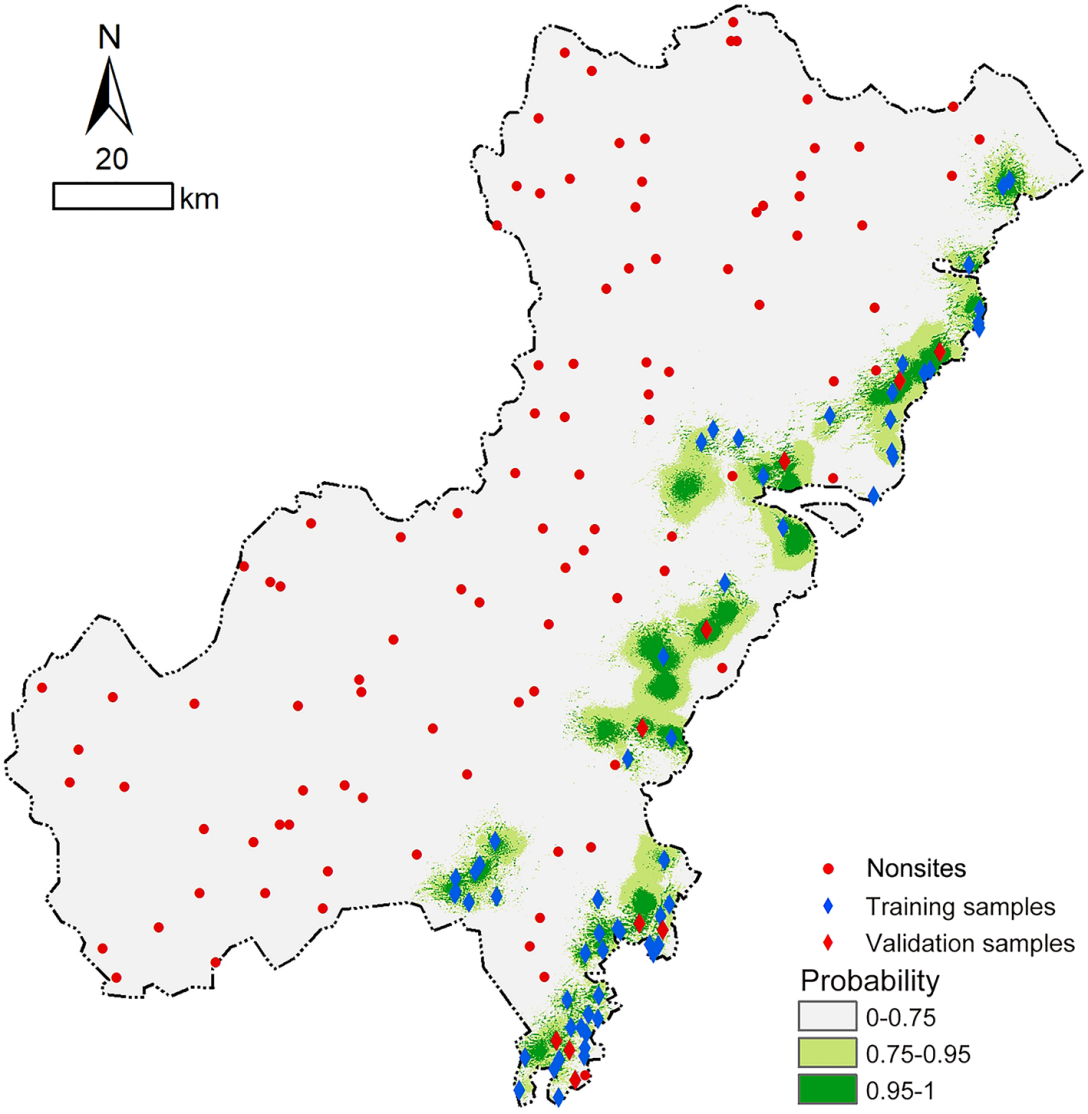
Locational probability map of beacon sites in the Wenzhou area (generated by the authors using ArcGIS 10.3.1).

The final locational probability map of the beacon sites in the Wenzhou area generated using Eq. (1) and it was classified into three probability classes: low (0–0.75), high (0.75–0.95) and very high (0.95–1) (Fig. 5). The extraction results of the probability values corresponding to the 10 validation samples, are shown in Table 4; the probability values corresponding to the 100 *nonsites* are shown in Supplementary Table S4. The data showed that all the 10 validation samples with probability values above 0.95, which were in the very high probability zone, indicated that the predictive model of beacon sites established in this study had a high efficiency and could be used for further research.

**Table 4:** Probability values of the validation samples.

Validation samples	1	2	3	4	5	6	7	8	9	10
Probability values	0.992	0.999	0.999	0.995	0.999	0.987	0.952	0.999	0.999	0.999

**Figure 6:**
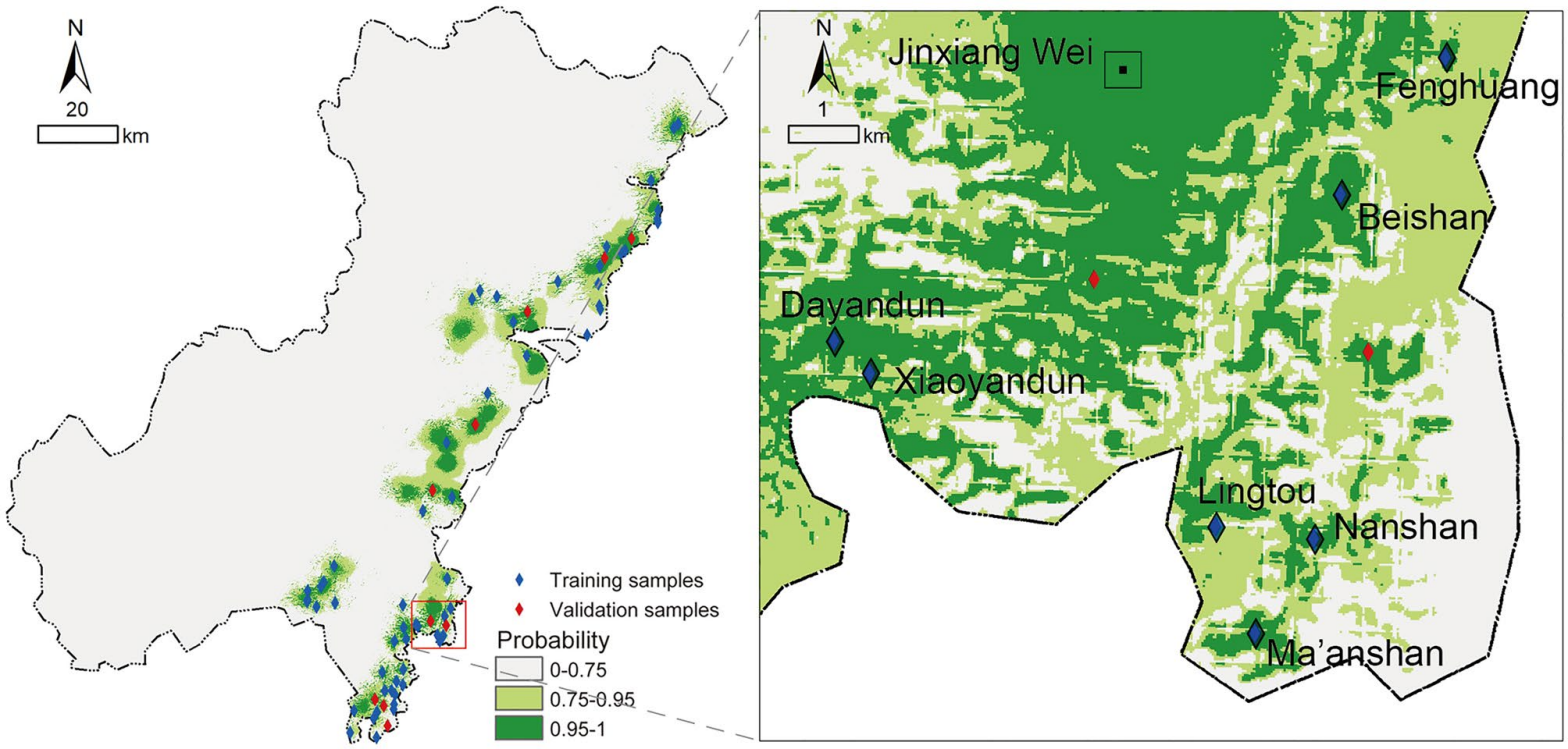
Viewshed analysis area (generated by the authors using ArcGIS 10.3.1).

Next, the viewshed analysis was conducted. Since the distribution of beacon towers in the northern area of Wenzhou was relatively simple, this study selected the beacon towers within the jurisdiction of Jinxiang Wei in the southern area as a case for analysis (Fig. 6). Jinxiang Wei was the highest-ranking fort in the southern area of Wenzhou, and the military mobilization and war command of other forts within this area were under its administration, which was the final collection point for all kinds of military information. There were seven known experimental beacon sites, two validation sites and one fort site in the study area. Taking each site point as the observation point, the visible conditions among the sites were calculated by viewshed analysis, and the statistical results are shown in Table 5.

**Table 5:** Viewshed analysis results statistics of experimental sites.

Sites		Jinxiang Wei	Fenghuang	Beishan	Lingtou	Nanshan	Ma’anshan	Xiaoyandun	Dayandun
Jinxiang Wei				·					
Fenghuang				·					
Beishan		·	·						
Lingtou						·	·		
Nanshan					·		·		
Ma’anshan					·	·			
Xiaoyandun									·
Dayandun								·	

· means visible.

**Figure 7:**
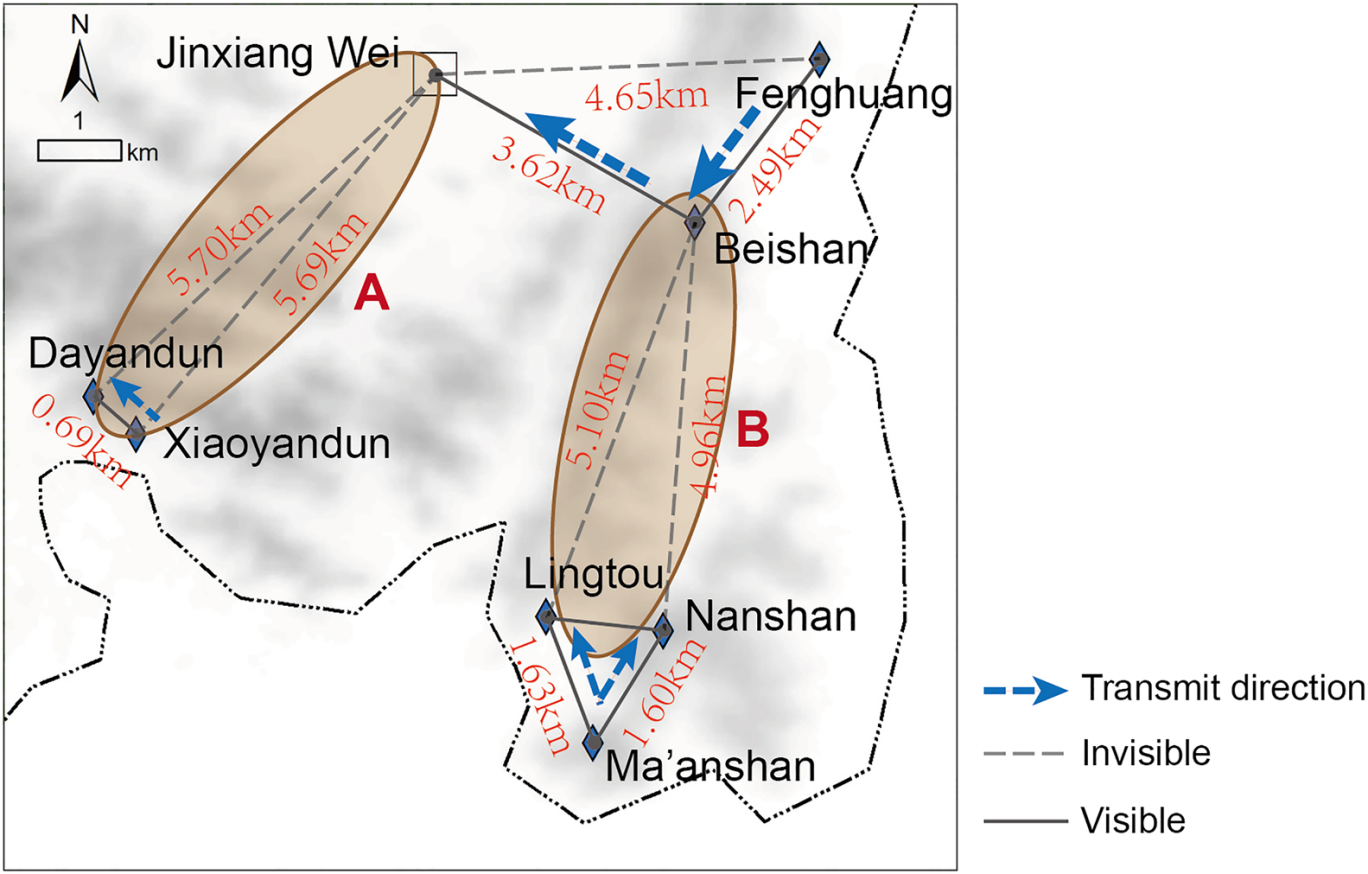
Viewshed analysis (generated by the authors using ArcGIS 10.3.1).

Through the preliminary viewshed analysis, it was found that the distance between the two beacon towers connected by sight is within 4 km, transmitting information to Jinxiang Wei from the southwest and southeast directions separately, which created a precise intelligence network. Among them, the Beishan beacon was within the visual area of Jinxiang Wei, and the Fenghuang beacon could also indirectly transmit information to Jinxiang Wei via the Beishan beacon. However, the transmission route between the Dayandun beacon and Jinxiang Wei and the transmission routes between the Lingtou beacon, Nanshan beacon and Beishan beacon were blocked so that the information from the Xiaoyandun beacon and Ma'anshan beacon could not be transmitted to Jinxiang Wei. Therefore, it was judged that other beacon towers that were not yet found might exist in areas A and B (Fig. 7).

**Figure 8:**
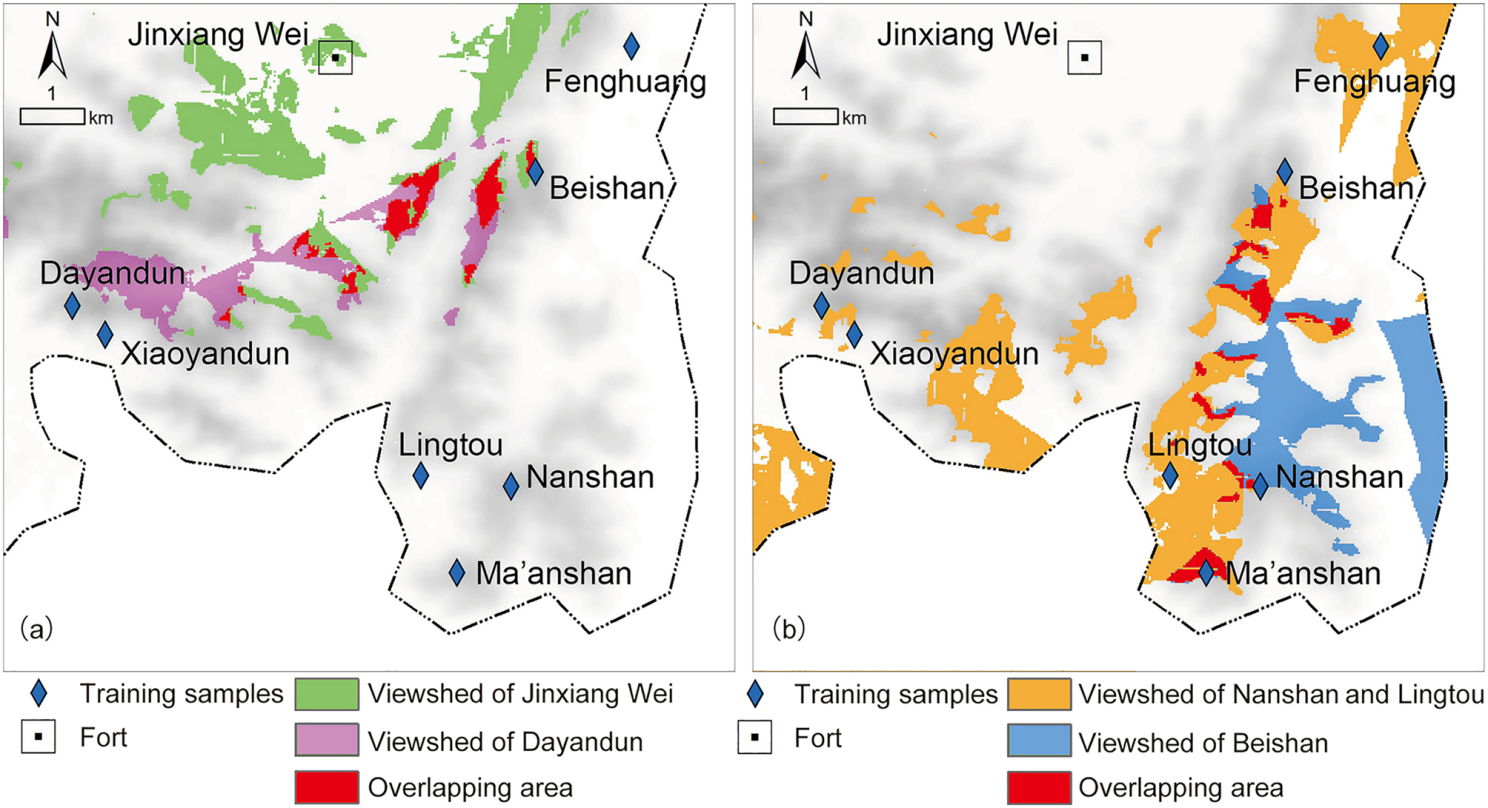
Viewshed analysis results: (**a**) the viewshed area of Jinxiang Wei and Dayandun Beacon; (**b**) the viewshed area of Nanshan Beacon, Lingtou Beacon and Beishan Beacon (generated by the authors using ArcGIS 10.3.1).

The viewshed areas of Jinxiang Wei and Dayandun Beacon (Fig. 8a) and the viewshed areas of Nanshan Beacon, Lingtou Beacon and Beishan Beacon (Fig. 8b) were superimposed to obtain the possible existence areas of beacon sites in terms of visual factors.

**Figure 9:**
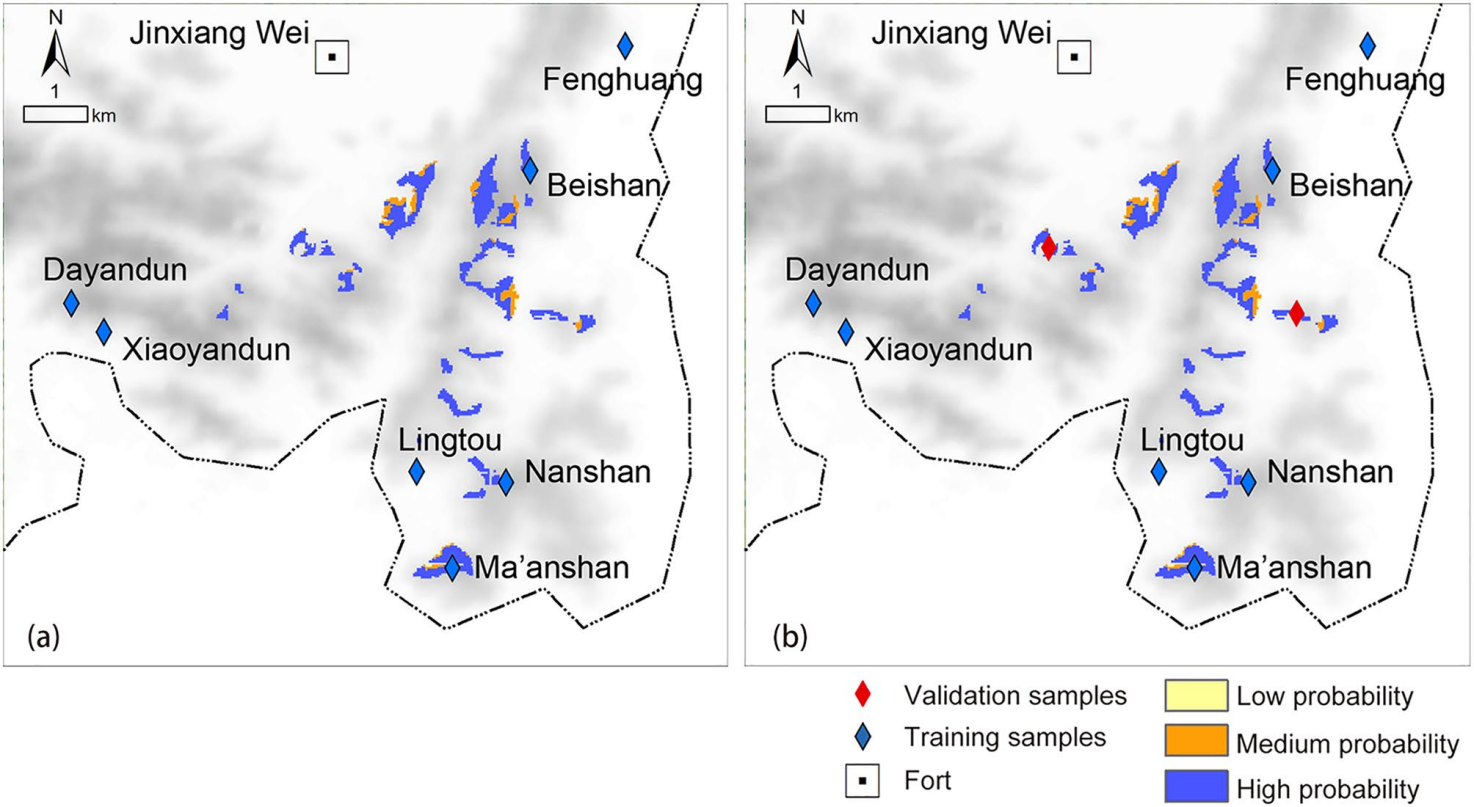
Final prediction result in the Jinxiang Wei area: (**a**) the prediction result; (**b**) validation result (generated by the authors using ArcGIS 10.3.1).

By superimposing the viewshed analysis result on the distribution probability map of the beacon sites (Fig. 6), the final prediction result of beacon sites in the Jinxiang Wei area was obtained (Fig. 9a). To verify the accuracy of the final prediction result, we projected the validation samples of beacon sites onto the final probability map (Fig. 9b), and it could be seen that the actual beacon site locations basically matched the final prediction result, which demonstrated the practicability of the predictive method constructed in this study.

In addition, the following conclusions can be drawn from this study: (1) by comparing the final prediction result obtained after superimposing viewshed analysis (Fig. 9b) with the single logistic regression prediction result (Fig. 6), it was found that the predictive scope is reduced by more than 90%, which greatly improved the accuracy and predictive ability of site prediction and can provide decision-making guidance for archaeological excavation work to a certain extent, thus effectively saving manpower and material resources, reducing the blindness of archaeological work, and yielding twice the result with half the effort; and (2) during the research process, the independent variables that have a significant influence on the establishment of the predictive model can be screened out through data extraction and statistical analysis, and all those variables have been regarded as important environmental factors that influence ancient humans’ residential choices by researchers in archaeological research. The results of GIS spatial analysis and logistic regression analysis suggested that the locational selection of beacon sites in Wenzhou was significantly influenced by traffic routes, followed by topographic relief and altitude, while slope did not have strong effects on site distribution. Moreover, the rules of siting for beacon towers in ancient times could also be inferred: the ancient people attached great importance to building beacon towers in areas with wide vision and small topographic relief, not only could get a good view, easy to look out but also convenient for construction; additionally, they also preferred locations near transportation system, where enemy activities were frequent, to monitor their movements and to quickly transmit alerts to military forts for timely decision making and counterattack organization.

Of course, the shortcomings of the method used in this study must be recognized: (1) the independent variables selected in this study are limited. The site selection of beacon towers might be closely related to military, politics, Fengshui, climate and other factors, which are difficult to extract quantitatively, so they are not considered influencing factors in this study; (2) there are some constraints in data acquisition. The research object was in ancient times, but ancient data could not be obtained, so modern data were used as a substitute; moreover, there were some human errors in data vectorization. If it is possible to obtain more accurate and reliable data, the prediction result will be more scientific and can further improve the reliability of the research; (3) the *nonsites* in the experimental samples come from the random points generated by ArcGIS software, among which are likely to be beacon sites waiting for excavation. When they act as *nonsites* in the predictive model, it will have a weak impact on the accuracy of the final prediction results. In addition, there might be some inevitable subjective factors when classifying the training and validation samples; and (4) there are certain limitations of the viewshed analysis method, which is only applicable to the case in which the beginning and ending beacon sites of a transmission route are known and the intermediate connection beacon site is missing. In contrast, it is impossible to make inferences by analyzing the viewshed of known beacon sites.

Nevertheless, the use of GIS and statistical methods has transformed archaeological research from qualitative to quantitative analysis, and the quantification of data analysis has provided new perspectives for settlement archaeological research. The predictive method of Wenzhou’s beacon sites proposed in this study has a huge breakthrough in prediction accuracy and ability compared with previous predictive methods, which can narrow the possible area of the beacon sites to a smaller scope and establish a predictive model with more practical predictive ability.

## Conclusions

In this study, 68 discovered beacon sites and 100 random *nonsites* in the Wenzhou area were taken as experimental samples, which were divided into two parts: training samples and validation samples. Data extraction, statistical analysis and screening of each independent variable were carried out using GIS spatial analysis tools, and the distribution predictive model of beacon sites was established through logistic regression. On this basis, the viewshed analysis of the beacon sites was conducted according to their visual attributes to precisely determine the predictive scope, improve the accuracy and predictive ability of traditional prediction methods, and reduce the difficulty of archaeological work to the greatest extent possible. The results showed that the predictive method of beacon sites proposed in this study reduced the possible location scope by more than 90% and was proven to be scientific and reliable.

From the perspective of historical research, the predictive model can also be used to investigate the relationship between the beacon sites and various natural and cultural environmental parameters, revealing the deployment characteristics of mountain beacon towers and deepening the understanding of military heritage. In addition, for heritage management, the site predictive model has great potential for application. China has numerous cultural heritages, and even though three national relic surveys have been conducted, many sites are still missing. In the face of frequent infrastructure construction, finding ways to improve efficiency, save costs, and protect the sites, that have not yet been discovered and registered, is a serious problem. The site predictive model can be used to predict the probability of site discovery in unknown locations and assist in protection and management decisions. Moreover, regional systematic investigation for settlement morphology research has been widely carried out nationwide, which provides the basic conditions for the establishment of archaeological site predictive models in various places. Therefore, the application and promotion of archaeological predictive models in heritage conservation and infrastructure construction are not only necessary but also practical.

## Supplementary Information

Supplementary Table S1.

Supplementary Table S2.

Supplementary Table S3.

Supplementary Table S4.

## Data Availability

All data generated or analyzed during this study are included in this published article (and its Supplementary Information files).
