# Supplementary material for: GIS-based precise predictive model of mountain beacon sites in Wenzhou, China
Source: Sci Rep. 2022 Jun 24;12:10773. doi: 10.1038/s41598-022-15067-z (PMC9232621; doi:10.1038/s41598-022-15067-z)
Supplement: Supplementary file 1 — Supplementary Table S1. [file 41598_2022_15067_MOESM1_ESM.pdf]

Supplementary Table S1 The attribute values of beacon sites for altitude, slope, aspect, topographic relief, distance from traffic routes, distance from nearest beacon tower or fort

Beacon sites	Altitude (m)	Slope (°)	Aspect*	Topographic relief (m)	Distance from traffic routes (m)	Distance from nearest beacon tower or fort (m)
1	216	8.95366478	183.5531464	205.7627716	2007.009888	2755.818196
2	633	11.67456627	52.55727386	622.963501	8802.349609	4136.305113
3	6	2.04785347	175.2211914	7.490776062	308.7900085	3630.023672
4	32	6.181671143	285.3703308	24.38960457	674.4932861	2044.955365
5	71	17.19173241	174.2419128	69.79290771	7382.419434	2472.512239
6	52	13.1503973	316.6524048	44.6269455	4599.630859	2472.512239
7	249	12.95741081	300.6141968	243.3857574	7734.419922	4125.420954
8	9	1.642006993	308.8607483	8.162238121	4341.409668	758.470197
9	84	8.320762634	122.8156815	79.27349854	749.241272	2112.427237
10	84	9.926810265	200.3490448	68.11196899	653.0274658	1210.530643
11	115	17.10284996	273.3693542	99.94002533	1575	3585.928474
12	103	5.298503399	167.1378021	97.45578003	1116.680054	1594.588797
13	113	3.572123289	242.6683807	104.5094986	1661.376099	3916.663214
14	105	7.838297367	264.0973206	93.89093018	2489.577148	957.6348598
15	79	7.21179533	219.3684845	69.37359619	2197.610107	2017.278848
16	252	7.856826782	80.11454773	239.9739075	4002.355957	4041.551096
17	113	10.71538448	239.5708771	97.48691559	7340.010742	834.6795389
18	118	6.862810135	255.0599365	105.3979568	6454.795898	834.6795389
19	218	8.596346855	92.55691528	209.2815094	5550.186035	8866.004406
20	28	3.42054987	233.223465	21.3287487	2641.361572	957.6348598
21	251	12.53694534	88.20761871	239.7532196	2338.382324	2473.170229
22	100	6.199034214	45.66908646	84.10559082	11666.30762	6435.417713
23	101	9.647629738	8.228553772	93.03801727	1008.650696	1277.560465
24	153	4.647305489	138.3202972	142.0020447	1274.9198	1277.560465
25	191	5.053994179	226.1346741	184.6947632	1871.307739	6728.90965
26	23	6.288214207	64.60155487	20.01465797	4037.370117	758.470197
27	26	14.23064709	21.86445618	21.4073925	134.7447205	4650.750919
28	329	12.78071785	253.103653	322.0725708	7216.524902	4694.167723
29	181	4.918808937	57.90693665	175.0246124	1746.018799	4650.750919
30	138	7.75078249	66.26986694	135.3988037	1433.341553	1202.31687
31	157	1.900163412	214.0323639	152.516922	2310.921143	439.2889961
32	128	14.1487999	293.5491943	129.633316	907.854187	2700.219453
33	31	9.465439796	169.8565674	29.44979858	5480.993164	2207.307644
34	282	8.004080772	34.72835159	271.9972839	4644.214355	2035.015393
35	104	28.08397865	187.5168457	101.7089157	2457.554932	610.6693118
36	264	25.10406685	240.2332001	253.1086121	938.8842163	610.6693118
37	43	13.38653374	135.6769104	35.03695297	308.789978	2502.14194
38	110	10.69120884	208.6784973	104.1473083	1715.39917	439.2889961

39	103	5.706999302	118.8471222	98.85942841	3823.313477	6693.001572
40	109	12.07091236	132.202774	94.38114166	2910.653564	4182.203899
41	151	7.736134052	85.45593262	146.336441	1804.334473	1202.31687
42	239	12.94900131	17.51441193	234.8930817	5336.463379	3107.860219
43	157	9.05950737	288.8327637	144.0826569	6766.391113	2035.015393
44	463	12.54863644	129.3594666	453.1763916	5457.774902	1595.76224
45	270	7.439590454	171.3312531	259.2945557	11136.16699	1110.838839
46	182	12.80293274	262.213562	176.9489899	10063.21973	1110.838839
47	146	8.121311188	285.7383728	135.0522156	7390.313477	1149.593087
48	262	8.622258186	15.35816193	259.605011	5299.811523	1516.41965
49	80	21.50418472	107.0844345	77.71052551	8565.996094	1595.76224
50	84	19.48274994	108.7642822	81.51739502	10319.65039	3670.20241
51	94	14.25580978	102.0594788	91.43635559	9707.177734	3625.165362
52	309	19.18732071	50.23556137	305.1193542	9498.613281	5212.308602
53	216	23.11184883	330.1951599	210.6345367	6010.686523	3820.776717
54	350	12.94978428	120.1443481	346.0115662	415.3660889	2708.804077
55	391	4.930762291	168.5438232	381.3019714	7039.804199	4265.580604
56	203	3.423363686	255.0206451	196.9971313	2543.544189	2424.50501
57	91	13.63427162	26.35578156	89.09870148	8042.583984	4967.135915
58	309	8.02377224	79.16446686	306.0101624	4731.209473	1567.412473
59	266	5.349405766	271.216156	254.7800598	8583.638672	1273.157879
60	341	4.522444725	236.9149933	330.194397	6088.068848	1614.676045
61	52	14.75576973	98.17712402	41.78478241	8254.588867	1149.593087
62	170	6.472849846	116.6425781	162.82547	6353.058105	2179.212902
63	125	29.13562965	152.0034027	129.7521057	1120.470337	2002.177565
64	419	6.901811123	109.9174805	411.8543396	6156.228027	2439.613747
65	47	14.60798931	129.5604248	45.81257629	6310.102051	1273.157879
66	213	17.57581711	318.0170593	204.9945221	8095.041992	1421.370615
67	255	14.88442326	34.78039169	239.3139496	2822.198486	2502.14194
68	3	0.723204076	63.44689941	4.496536255	4267.309082	1614.676045

(*: -1-0 means “Flat”; 0-22.5 and 337.5-360 means “North”; 22.5-67.5 means “Northeast”; 67.5-112.5 means “East”; 112.5-157.5 means “Southeast”; 157.5-202.5 means “South”; 202.5-247.5 means “Southwest”; 247.5-292.5 means “West”; 292.5-337.5 means “Northwest”.)
