# Supplementary material for: GIS-based precise predictive model of mountain beacon sites in Wenzhou, China
Source: Sci Rep. 2022 Jun 24;12:10773. doi: 10.1038/s41598-022-15067-z (PMC9232621; doi:10.1038/s41598-022-15067-z)
Supplement: Supplementary file 2 — Supplementary Table S2. [file 41598_2022_15067_MOESM2_ESM.pdf]

**Supplementary Table S2** The attribute values of nonsites for altitude, slope, aspect, topographic relief, distance from traffic routes, distance from nearest beacon tower or fort

| Nonsites | Altitude (m) | Slope (°) | Aspect* | Topographic relief (m) | Distance from traffic routes (m) | Distance from nearest beacon tower or fort (m) |
|---|---|---|---|---|---|---|
| 1 | 466 | 27.18008041 | 338.1063232 | 469.7755127 | 71763.32813 | 43616.88591 |
| 2 | 4 | 0 | -1 | 4.673469543 | 6056.074219 | 7871.106945 |
| 3 | 41 | 3.100763798 | 170.537674 | 41.93877411 | 8462.639648 | 7899.261497 |
| 4 | 651 | 23.20157242 | 222.473877 | 640.836731 | 44000.35156 | 22299.78431 |
| 5 | 58 | 24.88507462 | 176.6981354 | 61.04081726 | 3887.019287 | 17933.35842 |
| 6 | 357 | 25.28322029 | 2.161079407 | 355.0204163 | 21805.37109 | 17082.86311 |
| 7 | 236 | 9.75286293 | 253.4429321 | 239.020401 | 15512.15723 | 7205.715036 |
| 8 | 426 | 20.70110512 | 315.954834 | 430 | 8347.825195 | 4071.498476 |
| 9 | 1051 | 13.43969154 | 206.5650482 | 1037.204102 | 157635.6875 | 60294.23751 |
| 10 | 242 | 27.15905762 | 273.4832764 | 231.6530609 | 11718.18555 | 9450.852264 |
| 11 | 675 | 34.8670845 | 324.8880005 | 678.2041016 | 118456.4922 | 45977.03008 |
| 12 | 4 | 0.806739628 | 198.4349518 | 3.959183693 | 4923.567383 | 6663.301901 |
| 13 | 492 | 25.0295372 | 304.9024963 | 506.6122437 | 55857.65625 | 24769.33962 |
| 14 | 372 | 32.91028595 | 235.6539001 | 368.0816345 | 29194.82617 | 26407.8471 |
| 15 | 77 | 8.974905968 | 68.49856567 | 77.87754822 | 52994.49609 | 31537.29108 |
| 16 | 736 | 30.63604164 | 0.430787206 | 737.244873 | 120676.875 | 53137.25058 |
| 17 | 215 | 35.76695251 | 193.9503479 | 214.836731 | 26414.72461 | 19300.81401 |
| 18 | 887 | 26.4662056 | 79.69515228 | 874.3265381 | 41438.03516 | 37528.2339 |
| 19 | 103 | 11.08531475 | 180 | 100.0612259 | 6217.46875 | 7689.211437 |
| 20 | 252 | 32.82842636 | 186.3401947 | 251.9183655 | 2836.331543 | 26979.78048 |
| 21 | 521 | 27.13800621 | 288.7497559 | 519.918396 | 63397.45313 | 35139.14831 |
| 22 | 57 | 10.37345695 | 94.18491364 | 62.34693909 | 39059.55469 | 26807.91361 |
| 23 | 456 | 8.974905968 | 68.49856567 | 444.8775635 | 52054.71875 | 27402.57995 |
| 24 | 543 | 15.29137897 | 300.3236084 | 534.755127 | 91649.21875 | 44898.70661 |
| 25 | 292 | 7.178379536 | 225 | 286.5101929 | 5377.728516 | 31659.65898 |
| 26 | 392 | 27.45095825 | 165.6063995 | 391.7346802 | 39410.17578 | 18586.29147 |
| 27 | 361 | 7.266607761 | 245.2248535 | 359.9795837 | 26155.48242 | 58184.00118 |
| 28 | 791 | 16.82540131 | 283.6269836 | 787.163269 | 75129.07813 | 44768.02432 |
| 29 | 405 | 18.85549164 | 157.7824097 | 413.4898071 | 21854.49609 | 8447.105993 |
| 30 | 527 | 15.0848074 | 352.405365 | 531.081604 | 6590.724121 | 29979.45901 |
| 31 | 286 | 11.78144073 | 163.8865814 | 283.1836853 | 11478.55371 | 10703.41741 |
| 32 | 942 | 34.33150101 | 347.957428 | 949.6530762 | 54704.65625 | 27713.53825 |
| 33 | 810 | 22.41189384 | 232.0164948 | 818.2653198 | 22553.08398 | 31950.77218 |
| 34 | 132 | 27.91954422 | 0.481465816 | 137.6734619 | 7269.730469 | 17317.43188 |
| 35 | 7 | 7.178379536 | 315 | 10.34693909 | 1416.016724 | 9237.44883 |
| 36 | 294 | 17.80185699 | 19.44003487 | 283.3877563 | 41007.60938 | 24017.45414 |
| 37 | 680 | 24.29810143 | 67.98871613 | 675.836731 | 65628.48438 | 30900.33595 |
| 38 | 693 | 27.22728348 | 246.5409698 | 690.7142944 | 71856.46875 | 42974.69284 |

| | | | | | | |
|---|---|---|---|---|---|---|
| 39 | 280 | 4.999786377 | 284.7435608 | 283.3673401 | 16853.48047 | 59754.32969 |
| 40 | 641 | 34.1673317 | 19.14813805 | 634.7958984 | 113935.6172 | 45202.58695 |
| 41 | 78 | 13.98887348 | 151.1443329 | 77.12245178 | 990.4935303 | 35078.49401 |
| 42 | 393 | 12.69669342 | 71.56504822 | 387.2653198 | 17038.11523 | 18429.31082 |
| 43 | 554 | 19.59039116 | 121.7014313 | 549.1836548 | 39870.32031 | 19951.17177 |
| 44 | 908 | 22.9328537 | 120.3432465 | 906.5714111 | 18496.96094 | 28787.00612 |
| 45 | 848 | 24.79209709 | 340.8663635 | 840.3265381 | 35196.58984 | 26551.49138 |
| 46 | 47 | 15.82159233 | 188.1300964 | 56.32653046 | 19263.46875 | 10875.52449 |
| 47 | 127 | 28.18356133 | 195.4221649 | 129.8571472 | 8441.435547 | 11208.89377 |
| 48 | 294 | 15.91404057 | 104.4702911 | 292.8571472 | 14224.55664 | 25406.51298 |
| 49 | 310 | 15.7172699 | 355.4622192 | 305.0816345 | 11231.35742 | 4959.713921 |
| 50 | 972 | 35.72224426 | 352.170929 | 967.244873 | 78389.66406 | 32002.2956 |
| 51 | 8 | 6.906647205 | 162.8972778 | 9.673469543 | 15475.59082 | 10855.59647 |
| 52 | 828 | 34.98737717 | 14.7435627 | 836.1428833 | 140649.0469 | 56349.47184 |
| 53 | 379 | 7.895627022 | 95.52754211 | 388.040802 | 17099.24023 | 19837.65183 |
| 54 | 101 | 23.22841835 | 210.5559692 | 101.9387741 | 33611.95703 | 27169.07388 |
| 55 | 441 | 24.62296677 | 327.699585 | 451 | 18955.54883 | 13954.86985 |
| 56 | 364 | 15.74716187 | 291.2973633 | 364.3673401 | 18189.88477 | 5226.396451 |
| 57 | 16 | 7.319012642 | 303.6900635 | 17.7142849 | 6354.506836 | 20887.84778 |
| 58 | 747 | 19.11239243 | 115.9065094 | 751.6734619 | 26069.72266 | 19928.12921 |
| 59 | 678 | 12.43449402 | 313.3634338 | 682.6326294 | 51252.85547 | 55684.19352 |
| 60 | 615 | 20.59815979 | 256.2930298 | 614.8163452 | 41797.98047 | 21767.84745 |
| 61 | 466 | 18.40463066 | 105.5241089 | 465.4285583 | 62642.69531 | 28714.55715 |
| 62 | 554 | 17.54615974 | 0.806929469 | 550.2041016 | 15576.99316 | 14234.17632 |
| 63 | 193 | 47.57764053 | 326.9565735 | 184.1428528 | 3966.502441 | 9354.31231 |
| 64 | 355 | 17.41818047 | 263.4801941 | 368.6938782 | 14596.65723 | 47199.78058 |
| 65 | 140 | 10.02481937 | 310.9143982 | 130.9183655 | 871.9876709 | 10525.46146 |
| 66 | 202 | 19.10360718 | 315 | 209.5102081 | 54508.55469 | 28465.57496 |
| 67 | 265 | 7.667116165 | 145.7843018 | 258.7550964 | 6349.717285 | 51689.96283 |
| 68 | 65 | 30.65307426 | 59.77455139 | 66.57142639 | 14060.09375 | 20587.4355 |
| 69 | 113 | 15.13578701 | 327.0947571 | 111 | 51231.44141 | 31509.15844 |
| 70 | 743 | 13.48457718 | 254.9315186 | 744.8979492 | 50658.76563 | 54818.92381 |
| 71 | 662 | 27.5590992 | 154.7468414 | 666.5306396 | 113760.3828 | 47110.72342 |
| 72 | 761 | 10.97865295 | 189.246109 | 766.6326294 | 35317.84766 | 49900.7748 |
| 73 | 472 | 18.28424454 | 217.3331909 | 468.4489746 | 12962.95508 | 6977.180375 |
| 74 | 4 | 0 | -1 | 4 | 5639.361328 | 8912.336051 |
| 75 | 309 | 12.25117683 | 74.53878021 | 306.2857056 | 35567.8125 | 19701.24505 |
| 76 | 921 | 8.171840668 | 119.7448807 | 922.3673706 | 41673.01172 | 43226.39919 |
| 77 | 683 | 4.106805325 | 330.255127 | 685.3469238 | 39896.58984 | 51390.5503 |
| 78 | 649 | 31.1986599 | 270 | 639.7755127 | 77789.78906 | 46642.42942 |
| 79 | 647 | 10.69817829 | 261.8699036 | 641.5306396 | 97445.64063 | 37340.83796 |
| 80 | 78 | 22.20333481 | 96.89242554 | 76.44898224 | 12464.09473 | 18909.46246 |
| 81 | 221 | 18.29354095 | 175.3645325 | 224.2244873 | 5654.001465 | 8568.747844 |

| | | | | | | |
|---|---|---|---|---|---|---|
| 82 | 692 | 13.01838303 | 344.3577576 | 690.4285889 | 78187.53906 | 38381.44155 |
| 83 | 509 | 14.226964 | 329.3814087 | 506.3877563 | 77625.73438 | 28558.11017 |
| 84 | 967 | 31.55710411 | 119.5387802 | 966.8571167 | 83736.61719 | 53559.57352 |
| 85 | 280 | 29.76124191 | 289.5637817 | 281.4285583 | 5722.374512 | 22499.47774 |
| 86 | 699 | 13.81588078 | 185.1944275 | 700.1428833 | 97083.61719 | 44449.56261 |
| 87 | 560 | 13.95016766 | 165.4655457 | 564.1224365 | 53349.84375 | 68349.86078 |
| 88 | 171 | 8.967885017 | 196.3895416 | 175.4693909 | 25749.14844 | 26280.3694 |
| 89 | 5 | 1.803448081 | 8.130102158 | 4.591836929 | 4635.473633 | 2178.713694 |
| 90 | 624 | 16.62777328 | 20.05609512 | 619.2857056 | 63914.35938 | 38372.11809 |
| 91 | 670 | 46.25671387 | 145.7681427 | 661.9387817 | 32845.47266 | 59941.7153 |
| 92 | 190 | 28.04460907 | 308.2110291 | 200.1224518 | 22254.96094 | 28357.91644 |
| 93 | 635 | 12.91059685 | 119.0546036 | 636.5714111 | 96494.85156 | 52445.84842 |
| 94 | 980 | 15.34154129 | 76.86597443 | 991.9387817 | 57185.08984 | 26329.08207 |
| 95 | 5 | 0.806739628 | 71.56504822 | 4.714285851 | 1653.758057 | 4773.233947 |
| 96 | 629 | 16.4732151 | 18.43494797 | 630.2653198 | 45030.76953 | 19282.60545 |
| 97 | 196 | 34.77351379 | 96.2590332 | 190.5510254 | 1790.876953 | 33788.63764 |
| 98 | 895 | 27.82554245 | 170.7760773 | 888.1020508 | 36125.39453 | 38622.21306 |
| 99 | 0 | 0 | -1 | 0 | 10992.43652 | 2868.37244 |
| 100 | 389 | 34.82091522 | 92.93567657 | 397.3061218 | 67142.38281 | 44300.62318 |

(*: -1-0 means “Flat”; 0-22.5 and 337.5-360 means “North”; 22.5-67.5 means “Northeast”; 67.5-112.5 means “East”; 112.5-157.5 means “Southeast”; 157.5-202.5 means “South”; 202.5-247.5 means “Southwest”; 247.5-292.5 means “West”; 292.5-337.5 means “Northwest”. )
