# Supplementary material for: GIS-based precise predictive model of mountain beacon sites in Wenzhou, China
Source: Sci Rep. 2022 Jun 24;12:10773. doi: 10.1038/s41598-022-15067-z (PMC9232621; doi:10.1038/s41598-022-15067-z)
Supplement: Supplementary file 3 — Supplementary Table S3. [file 41598_2022_15067_MOESM3_ESM.pdf]

Supplementary Table S3 Data for logistic regression

X1	X2	X3	X4	X5	Site or not*
216	7.106974602	205.4081573	2007.009888	8093.563628	1
631	12.25117683	621.836731	8802.349609	12033.40628	1
32	6.555387974	24.38775444	674.4932861	12214.50335	1
70	17.09464836	77.89795685	7382.419434	2472.512239	1
55	11.09647655	48.24489975	4599.630859	2472.512239	1
249	13.52929211	244.3469391	7734.419922	4125.420954	1
9	1.442929983	8.265306473	4341.409668	758.470197	1
84	9.75286293	68.30612183	749.241272	4041.551096	1
116	16.61379623	100.7959213	653.0274658	6745.605675	1
104	9.162307739	93.32653046	1575	957.6348598	1
76	12.07488346	66.97959137	2489.577148	2017.278848	1
252	8.10958004	239.7550964	2197.610107	4041.551096	1
116	3.76154089	100.18367	4002.355957	834.6795389	1
119	4.802510738	106.163269	7340.010742	834.6795389	1
219	7.895627022	210.979599	6454.795898	9031.235774	1
28	2.624889612	21.67346954	5550.186035	957.6348598	1
247	16.16949081	237.9183655	2641.361572	3571.390381	1
100	5.348220825	84.38775635	2338.382324	6435.417713	1
100	10.63994312	92.4897995	11666.30762	1277.560465	1
153	4.215535641	142.836731	1008.650696	1277.560465	1
191	5.583516121	184	1274.9198	6728.90965	1
23	5.408027649	19.55101967	1871.307739	758.470197	1
19	15.79558945	18.10204124	4037.370117	7273.799866	1
327	13.15746403	320.795929	134.7447205	7273.799866	1
139	9.590919495	136.2040863	7216.524902	1202.31687	1
158	1.020413518	152.1020355	1433.341553	439.2889961	1
34	10.22823524	31.53061295	2310.921143	2207.307645	1
282	8.804753304	271.7755127	5480.993164	2207.307645	1
111	33.14856339	106.7755127	4644.214355	610.6693118	1
260	26.48448181	252.7550964	2457.554932	610.6693118	1
113	9.623542786	105.408165	938.8842163	439.2889961	1
100	6.860269547	97.55101776	1715.39917	6693.001572	1
108	13.28121471	93.81632996	3823.313477	4182.203899	1
151	7.465408802	147.3061218	2910.653564	1202.31687	1
236	15.0848074	233.3877563	1804.334473	3107.860219	1
465	11.0685482	454.4693909	5336.463379	1595.76224	1
270	7.863409519	259.040802	5457.774902	1110.838839	1
180	15.21382046	174.6326599	11136.16699	1110.838839	1
145	10.20992374	134.2040863	10063.21973	1149.593087	1
261	8.171840668	259.0204163	7390.313477	1516.41965	1
76	21.29949951	74.57142639	5299.811523	1595.76224	1

81	19.45090485	77.7755127	8565.996094	4345.086878	1
305	21.81438446	302.836731	10319.65039	5212.308602	1
214	24.5160923	209.163269	9498.613281	3820.776717	1
348	13.01838303	344.2857056	6010.686523	2708.804077	1
391	3.709561825	383.3265381	415.3660889	4265.580604	1
92	13.76349163	87.02040863	7039.804199	4967.135915	1
309	8.015232086	305.5714417	8042.583984	4729.647158	1
266	5.490637779	255.18367	4731.209473	1273.157879	1
341	4.842621326	329.5510254	8583.638672	1614.676045	1
52	15.0848074	41.61224365	6088.068848	1149.593087	1
125	29.23799896	130.0816345	8254.588867	2002.177565	1
418	8.171840668	411.163269	1120.470337	2439.613747	1
46	13.00438213	45.63265228	6156.228027	1273.157879	1
213	17.88500023	205.4897919	6310.102051	1421.370615	1
252	16.94193077	236.5306091	8095.041992	2502.14194	1
3	0.360804021	4.530612469	2822.198486	1614.676045	1
41	13.86804199	33.16326523	4267.309082	2502.14194	1
466	27.18008041	469.7755127	71763.32813	43616.88591	0
4	0	4.673469543	6056.074219	7871.106945	0
41	3.100763798	41.93877411	8462.639648	7899.261497	0
651	23.20157242	640.836731	44000.35156	22299.78431	0
58	24.88507462	61.04081726	3887.019287	17933.35842	0
357	25.28322029	355.0204163	21805.37109	17082.86311	0
236	9.75286293	239.020401	15512.15723	7205.715036	0
426	20.70110512	430	8347.825195	4071.498476	0
1051	13.43969154	1037.204102	157635.6875	60294.23751	0
242	27.15905762	231.6530609	11718.18555	9450.852264	0
675	34.8670845	678.2041016	118456.4922	45977.03008	0
4	0.806739628	3.959183693	4923.567383	6663.301901	0
492	25.0295372	506.6122437	55857.65625	24769.33962	0
372	32.91028595	368.0816345	29194.82617	26407.8471	0
77	8.974905968	77.87754822	52994.49609	31537.29108	0
736	30.63604164	737.244873	120676.875	53137.25058	0
215	35.76695251	214.836731	26414.72461	19300.81401	0
887	26.4662056	874.3265381	41438.03516	37528.2339	0
103	11.08531475	100.0612259	6217.46875	7689.211437	0
252	32.82842636	251.9183655	2836.331543	26979.78048	0
521	27.13800621	519.918396	63397.45313	35139.14831	0
57	10.37345695	62.34693909	39059.55469	26807.91361	0
456	8.974905968	444.8775635	52054.71875	27402.57995	0
543	15.29137897	534.755127	91649.21875	44898.70661	0
292	7.178379536	286.5101929	5377.728516	31659.65898	0
392	27.45095825	391.7346802	39410.17578	18586.29147	0

361	7.266607761	359.9795837	26155.48242	58184.00118	0
791	16.82540131	787.163269	75129.07813	44768.02432	0
405	18.85549164	413.4898071	21854.49609	8447.105993	0
527	15.0848074	531.081604	6590.724121	29979.45901	0
286	11.78144073	283.1836853	11478.55371	10703.41741	0
942	34.33150101	949.6530762	54704.65625	27713.53825	0
810	22.41189384	818.2653198	22553.08398	31950.77218	0
132	27.91954422	137.6734619	7269.730469	17317.43188	0
7	7.178379536	10.34693909	1416.016724	9237.44883	0
294	17.80185699	283.3877563	41007.60938	24017.45414	0
680	24.29810143	675.836731	65628.48438	30900.33595	0
693	27.22728348	690.7142944	71856.46875	42974.69284	0
280	4.999786377	283.3673401	16853.48047	59754.32969	0
641	34.1673317	634.7958984	113935.6172	45202.58695	0
78	13.98887348	77.12245178	990.4935303	35078.49401	0
393	12.69669342	387.2653198	17038.11523	18429.31082	0
554	19.59039116	549.1836548	39870.32031	19951.17177	0
908	22.9328537	906.5714111	18496.96094	28787.00612	0
848	24.79209709	840.3265381	35196.58984	26551.49138	0
47	15.82159233	56.32653046	19263.46875	10875.52449	0
127	28.18356133	129.8571472	8441.435547	11208.89377	0
294	15.91404057	292.8571472	14224.55664	25406.51298	0
310	15.7172699	305.0816345	11231.35742	4959.713921	0
972	35.72224426	967.244873	78389.66406	32002.2956	0
8	6.906647205	9.673469543	15475.59082	10855.59647	0
828	34.98737717	836.1428833	140649.0469	56349.47184	0
379	7.895627022	388.040802	17099.24023	19837.65183	0
101	23.22841835	101.9387741	33611.95703	27169.07388	0
441	24.62296677	451	18955.54883	13954.86985	0
364	15.74716187	364.3673401	18189.88477	5226.396451	0
16	7.319012642	17.7142849	6354.506836	20887.84778	0
747	19.11239243	751.6734619	26069.72266	19928.12921	0
678	12.43449402	682.6326294	51252.85547	55684.19352	0
615	20.59815979	614.8163452	41797.98047	21767.84745	0
466	18.40463066	465.4285583	62642.69531	28714.55715	0
554	17.54615974	550.2041016	15576.99316	14234.17632	0
193	47.57764053	184.1428528	3966.502441	9354.31231	0
355	17.41818047	368.6938782	14596.65723	47199.78058	0
140	10.02481937	130.9183655	871.9876709	10525.46146	0
202	19.10360718	209.5102081	54508.55469	28465.57496	0
265	7.667116165	258.7550964	6349.717285	51689.96283	0
65	30.65307426	66.57142639	14060.09375	20587.4355	0
113	15.13578701	111	51231.44141	31509.15844	0

743	13.48457718	744.8979492	50658.76563	54818.92381	0
662	27.5590992	666.5306396	113760.3828	47110.72342	0
761	10.97865295	766.6326294	35317.84766	49900.7748	0
472	18.28424454	468.4489746	12962.95508	6977.180375	0
4	0	4	5639.361328	8912.336051	0
309	12.25117683	306.2857056	35567.8125	19701.24505	0
921	8.171840668	922.3673706	41673.01172	43226.39919	0
683	4.106805325	685.3469238	39896.58984	51390.5503	0
649	31.1986599	639.7755127	77789.78906	46642.42942	0
647	10.69817829	641.5306396	97445.64063	37340.83796	0
78	22.20333481	76.44898224	12464.09473	18909.46246	0
221	18.29354095	224.2244873	5654.001465	8568.747844	0
692	13.01838303	690.4285889	78187.53906	38381.44155	0
509	14.226964	506.3877563	77625.73438	28558.11017	0
967	31.55710411	966.8571167	83736.61719	53559.57352	0
280	29.76124191	281.4285583	5722.374512	22499.47774	0
699	13.81588078	700.1428833	97083.61719	44449.56261	0
560	13.95016766	564.1224365	53349.84375	68349.86078	0
171	8.967885017	175.4693909	25749.14844	26280.3694	0
5	1.803448081	4.591836929	4635.473633	2178.713694	0
624	16.62777328	619.2857056	63914.35938	38372.11809	0
670	46.25671387	661.9387817	32845.47266	59941.7153	0
190	28.04460907	200.1224518	22254.96094	28357.91644	0
635	12.91059685	636.5714111	96494.85156	52445.84842	0
980	15.34154129	991.9387817	57185.08984	26329.08207	0
5	0.806739628	4.714285851	1653.758057	4773.233947	0
629	16.4732151	630.2653198	45030.76953	19282.60545	0
196	34.77351379	190.5510254	1790.876953	33788.63764	0
895	27.82554245	888.1020508	36125.39453	38622.21306	0
0	0	0	10992.43652	2868.37244	0
389	34.82091522	397.3061218	67142.38281	44300.62318	0

(*: "1" means beacon site, "0" means nonsite.)
