# Supplementary material for: GIS-based precise predictive model of mountain beacon sites in Wenzhou, China
Source: Sci Rep. 2022 Jun 24;12:10773. doi: 10.1038/s41598-022-15067-z (PMC9232621; doi:10.1038/s41598-022-15067-z)
Supplement: Supplementary file 4 — Supplementary Table S4. [file 41598_2022_15067_MOESM4_ESM.pdf]

Supplementary Table S4 The probability values of nonsitesn

Nonsites	Probability values
1	0.000
2	0.006
3	0.013
4	0.000
5	0.000
6	0.000
7	0.000
8	0.059
9	0.000
10	0.002
11	0.000
12	0.164
13	0.000
14	0.000
15	0.000
16	0.000
17	0.000
18	0.000
19	0.063
20	0.000
21	0.000
22	0.000
23	0.000
24	0.000
25	0.000
26	0.000
27	0.000
28	0.000
29	0.000
30	0.000
31	0.000
32	0.000
33	0.000
34	0.000
35	0.021
36	0.000
37	0.000
38	0.000
39	0.000
40	0.000
41	0.000

42	0.000
43	0.000
44	0.000
45	0.000
46	0.000
47	0.005
48	0.000
49	0.004
50	0.000
51	0.000
52	0.000
53	0.000
54	0.000
55	0.000
56	0.000
57	0.000
58	0.000
59	0.000
60	0.000
61	0.000
62	0.000
63	0.333
64	0.000
65	0.856
66	0.000
67	0.000
68	0.000
69	0.000
70	0.000
71	0.000
72	0.000
73	0.002
74	0.218
75	0.000
76	0.000
77	0.000
78	0.000
79	0.000
80	0.000
81	0.004
82	0.000
83	0.000
84	0.000

85	0.000
86	0.000
87	0.000
88	0.000
89	0.667
90	0.000
91	0.000
92	0.000
93	0.000
94	0.000
95	0.728
96	0.000
97	0.000
98	0.000
99	0.433
100	0.000
